# Honeysuckle-Derived miR2911 Inhibits Replication of Porcine Reproductive and Respiratory Syndrome Virus by Targeting Viral Gene Regions

**DOI:** 10.3390/v16091350

**Published:** 2024-08-23

**Authors:** Xinyan Cao, Jiaxi Xue, Adnan Ali, Manyi Zhang, Jinliang Sheng, Yanming Sun, Yanbing Zhang

**Affiliations:** 1 College of Animal Science and Technology, Shihezi University, Shihezi 832003, China; caoxinyan12@outlook.com (X.C.); 19190249702@163.com (J.X.); adnanpak721@gmail.com (A.A.); jinliangsheng@shzu.edu.cn (J.S.); 2 Xinjiang Production & Construction Corps Key Laboratory of Animal Biomedicine, Tumushuke 843900, China; 13466324504@163.com

**Keywords:** miR2911, viral gene, target region, miRanda, honeysuckle, antiviral

## Abstract

The highly abundant and stable antiviral small RNA derived from honeysuckle, known as miR2911, has been shown to play a key role in inhibiting influenza virus infection and SARS-CoV-2 infection. However, whether miR2911 inhibits the replication of porcine reproductive and respiratory syndrome virus (PRRSV) remains unknown. Hence, this study investigated the mechanisms underlying the action of miR2911 during PRRSV infection. Six targets of miR2911 within the PRRSV orf1 (Nsp2: 2459 to 2477, 1871 to 1892, 954 to 977, and 1271 to 1292; Nsp1: 274 to 296 and 822 to 841) were successfully identified by using the miRanda v1.0b software. The miR2911 target sequence was analyzed by target sequence comparison, and only individual base mutations existed in different prevalent strains, and the miR2911 target region was highly conserved among different strains. Subsequently, through the dual luciferase reporter gene assay and miR2911 overexpression assay, it was demonstrated that miR2911 significantly inhibits the replication of PRRSV by targeting regions of PRRSV Nsp1 and Nsp2. These findings offer new insights for the development of novel anti-PRRSV drugs.

## 1. Introduction

MicroRNAs (miRNAs) are a class of highly conserved non-coding RNAs, typically 22 nucleotides in length [1,2]. The maturation process of miRNAs involves several distinct stages. Firstly, pri-miRNA is transcribed in the nucleus, which is then processed by the RNase III enzyme Drosha enzyme to yield pre-miRNA. Subsequently, the pre-miRNA is transported out of the nucleus into the cytoplasm for further maturation [3,4,5,6,7]. The function of miRNAs within the RNA-induced silencing complex (RISC) results in two distinct outcomes based on the complementarity of the miRNA guide strand to the 3′UTR of the target mRNA. In cases of incomplete complementarity, translation of the target gene is blocked and its expression is inhibited. Conversely, complete complementarity leads to cleavage and subsequent degradation of the target mRNA by the AGO2 protein [8]. miRNAs exhibit diverse sequences and structures, and exert critical regulatory functions in biological processes and protein synthesis. They have significant implications in disease control, prevention, and management, as well as in the development of the human genome. Their roles extend to influencing biological regulation and protein synthesis, including impacting the proliferation and apoptosis of diseased cells, cancer therapy, and mitigating toxic infections [9,10].

Honeysuckle is an important traditional Chinese medicine that is widely used in the treatment of influenza viruses and other infections. In 2015, it was demonstrated that mice infused with honeysuckle soup exhibited higher abundance of miR2911, which effectively targeted a broad spectrum of influenza A viruses (IAVs). Furthermore, chemically synthesized miR2911 not only significantly inhibited the expression of H1N1 PB2 and SN1 proteins but also reduced the mortality of H5N1-infected mice [11]. Additionally, Zhou et al. [12] found that miR2911 can effectively inhibit the replication of SARS-CoV-2 and has multiple inhibitory targets in the SARS-CoV-2 gene region. This suggests that honeysuckle miR2911, as an herbal medicine, may possess a wider range of antiviral effects.

PRRSV is a single-stranded positive-sense RNA virus with a capsular membrane belonging to the family *Arteriviridae* [13], and mainly infects macrophages and clinically induces acute respiratory disease in piglets as well as reproductive disorders in sows. PRRSV belongs to the genus *Betaarterivirus suid* in the family *Arteriviridae* and is a single-stranded positive-sense RNA virus with a capsid [14]. The PRRSV genome is approximately 15 kb long and contains a 5′UTR, at least 10 open reading frames (ORFs), a 3′-UTR, and a 3′poly (A) tail [15,16]. PRRSV has two genotypes: PRRSV-1 (*Betaarterivirus suid 1*) of European origin and PRRSV-2 (*Betaarterivirus suid 2*) of North American origin [13]. Porcine reproductive and respiratory syndrome (PRRS) was first identified in the United States in 1987. In 2006, HP-PRRSV strains broke out in several provinces of China, leading to severe economic losses. After 2012, recombinant strains such as neo-GM2 and NADC30-like appeared in China, with NADC30-like viruses becoming prevalent after 2015 [16]. The emergence of NADC30-like strains may be attributed to the recombination of North American NADC30 strains and Chinese HP-PRRSV strains. Although not as highly pathogenic as HP-PRRSV, NADC30-like strains are known for their high rate of recombination with other viral strains, resulting in altered virulence. Additionally, available vaccines do not provide complete protection against attacks by mutated strains, and outbreaks of NADC30-like strains may occur in vaccinated pigs [12,13,17,18].

Therefore, there is an urgent need to develop drugs that can treat or prevent mutated strains of PRRSV. Traditional Chinese herbs in China have shown effectiveness in the prevention and treatment of viral infectious diseases. Considering the powerful antiviral effect of honeysuckle-derived miR2911, this paper explores the role of honeysuckle-derived miR2911 in PRRSV infection through bioinformatics prediction, experimental validation, and other methods to provide new ideas for the development of novel antiviral drugs.

## 2. Materials and Methods

### 2.1. Materials

The following cell culture reagents were used in this study: DMEM medium (HyClone), fetal bovine serum (Gibco™), PBS (HyClone), dual luciferase activity assay kit (Novozymes Bio), pGL3-promoter plasmid (a gift from researcher Qiu Yafeng), and pGL3-promoter-PRRSV plasmid synthesized by Sangyo Biotech (Zhengzhou, China). MARC-145 cells and HEK-293T cells were cultured with DMEM medium containing 10% fetal bovine serum (FBS) and antibiotics (100 U/mL penicillin and 100 U/mL streptomycin).

### 2.2. Methods

#### 2.2.1. Honeysuckle miR2911 Sequence Acquisition

The mature sequence of honeysuckle-derived miR2911: 5′-CGGCCGGGGGACGGGCUGGG-3′ (Accession: GSE55268).

#### 2.2.2. Download of PRRSV Whole Gene Sequence

Enter the NCBI database, search for the full gene sequence of PRRSV, and choose to download the full gene sequence of the epidemic strains (MN119305.1).

#### 2.2.3. Prediction of PRRSV Gene Regions Targeted by miR2911

The analysis of miR2911 targeting PRRSV gene regions was performed by the online version of miRanda (http://www.bioinformatics.com.cn/local_miranda_miRNA_target_prediction_120 (accessed 25 October 2022)) database.

#### 2.2.4. Evolutionary Tree Construction of miR2911 Targeting Regions

MiR2911 targeting PRRSV gene regions was compared, and PRRSV sequences of different prevalent strains were downloaded to splice in the same order and construct an evolutionary tree using the neighbor-joining method implemented in MEGA 7.0.26 software.

#### 2.2.5. Target Region Validation

HEK-293T cells were co-transfected with miR2911 mimics (Table S1) along with the pGL3-promoter-PRRSV plasmid (Table S2) and pRL-TK. The miRNA negative control (NC) (Table S3) was co-transfected with the pGL3-promoter-PRRSV plasmid to serve as a control. Following transfection, Vazyme Lipomaster 3000 Transfection Reagent (TL301-01, Nanjing, China) was utilized to transfect the cells with the pGL3-promoter-PRRSV plasmid and miRNA. After transfection for 24 h, the cells were analyzed according to the instructions of the Vazyme Dual Luciferase Reporter Gene Assay Kit (DD1205-01, Nanjing, China). Differential analysis was conducted by plotting graphs using GraphPad Prism 5.0.

#### 2.2.6. miR2911 Antiviral Analysis

MARC-145 cells were inoculated in 24-well plates, cultured overnight, and then transfected with miR2911, with miRNA NC transfected as a control. Twelve hours after transfection, the cells were infected with PRRSV at 0.1 MOI, and the expression of viral orf7 and viral titer was determined 24 h after infection.

Monkey β-actin was used as the internal reference gene, and orf7 of PRRSV was considered the target gene [13] (Table S4). Total RNA was extracted from cell samples, and cDNA was synthesized according to the instructions of the reverse transcription kit. The cDNA was then used as the template to amplify the orf7 and β-actin genes, and RT-qPCR was performed according to the instructions of SYBR^®^ Premix (Yeasen Biotechnology Company). Three replicates were set up for each group of samples, and the obtained data were calculated and analyzed using the 2-ΔΔCt method. Graphs were plotted using GraphPad Prism 5.0 for differential analysis.

#### 2.2.7. Statistical Analysis

All data were analyzed with GraphPad Prism Version 5.01 software (GraphPad Software, San Diego, CA, USA). An unpaired Student’s *t*-test was used to determine the significant differences. Values were considered statistically significant when *p* < 0.05 or *p* < 0.01. Data are presented as mean ± SEM, as indicated.

## 3. Results

### 3.1. Analysis of miR2911 Targeting PRRSV Gene Region

**Figure 1:**
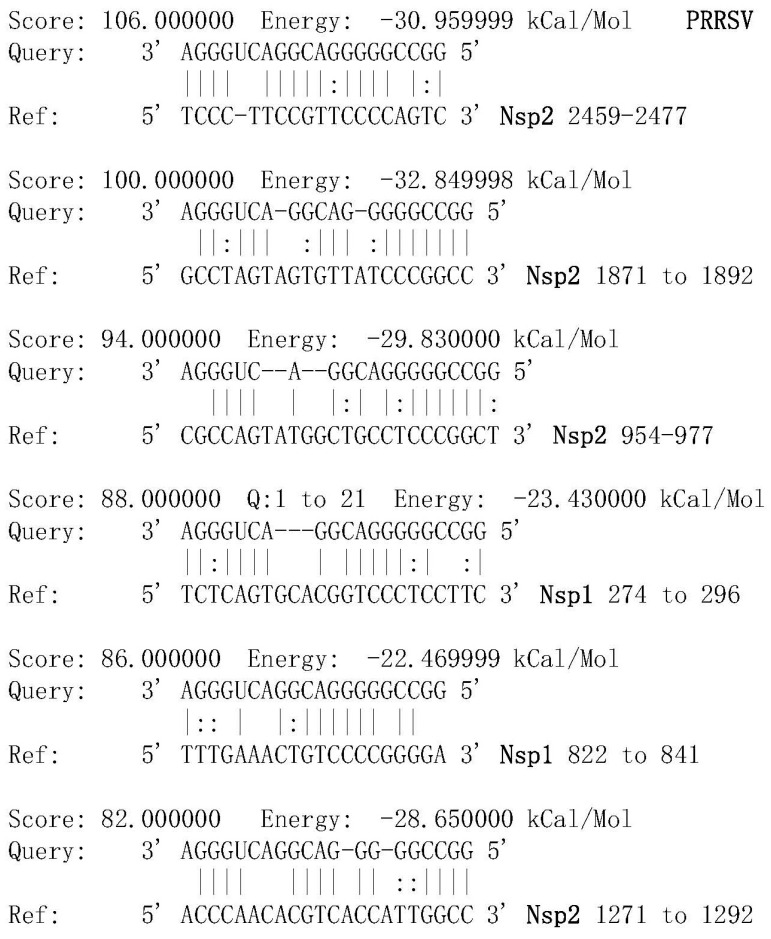
Prediction of miR2911 targeting PRRSV gene analysis (Note: “:” indicates base mismatch). Prediction of miR2911 targeting four regions of the PRRSV Nsp2 gene versus two regions of the Nsp1 gene using miRanda v1.0b software.

Using the miRanda v1.0b software prediction, it was identified that miR2911 targets six regions of the PRRSV orf1, including four regions of PRRSV Nsp2 and two regions of PRRSV Nsp1 (Figure 1). miR2911 can target the regions of PRRSV Nsp2 (2459 to 2477, 1871 to 1892, 954 to 977, and 1271 to 1292), with scores ranging from 82 to 106 and free energy ranging from −30.95 to −28.65 kCal/Mol. Additionally, miR2911 can target the regions of PRRSV Nsp1 (274 to 296 and 822 to 841), with scores ranging from 82 to 88 and free energy ranging from −23.43 to −22.47 kCal/Mol.

### 3.2. Sequence Evolution Tree Analysis of miR2911 Targeting PRRSV Gene Region

**Figure 2:**
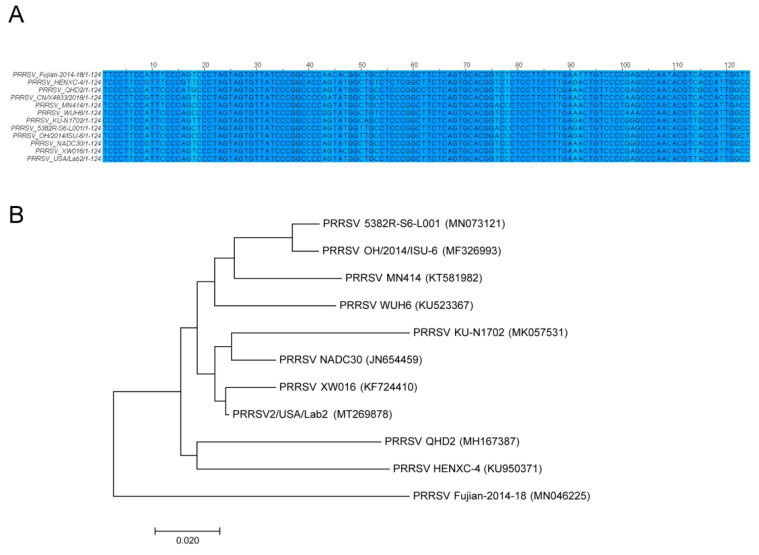
Evolutionary tree analysis of miR2911 targeting viral regions, targeting PRRSV genes with multi-region sequence evolutionary trees. (**A**) miR2911 sequences downloaded from the NCBI database targeting the PRRSV gene region, with light colors indicating genes with differences. (**B**) Construction of an evolutionary tree of miR2911 targeting viral gene regions using the neighborhood method in MEGA 7.0 software.

The sequences of miR2911 targeting PRRSV gene regions were connected in series according to the sequence, and the evolution tree of miR2911 targeting viral gene regions was constructed by using the MEGA 7.0 software. There were only two to four base mutations in the different strains (Figure 2A). The homology of miR2911 targeting PRRSV gene regions was more than 80% (Figure 2B).

### 3.3. miR2911 Significantly Inhibited PRRSV Replication via Targeted PRRSV Orf1

**Figure 3:**
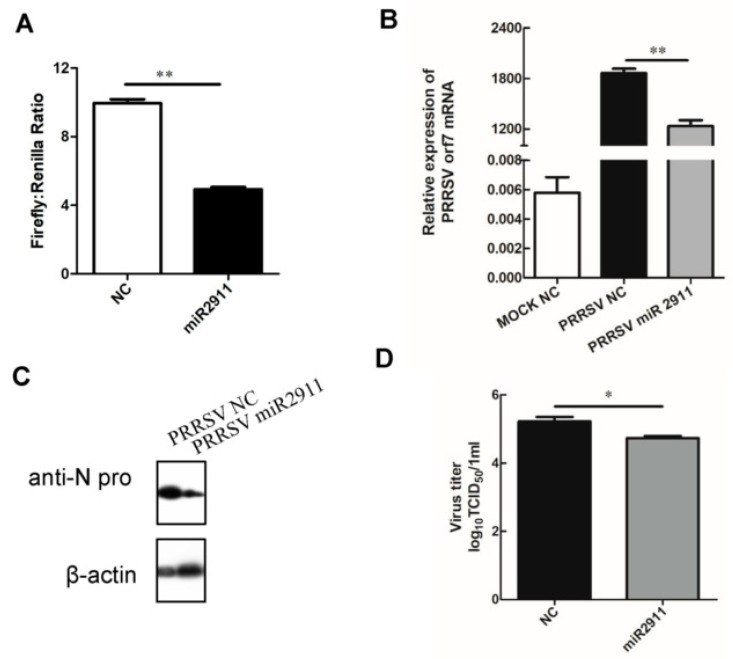
Overexpression of miR2911 inhibited replication of PRRSV via target viral gene regions. (**A**) HEK293T cells were co-transfected with luciferase reporter pGL3-promoter-PRRSV, pRL-TK, and miR2911 mimic or NC mimic; after transfection for 24 h, double luciferase reporter was detected. (**B**,**C**) miR2911 or NC was transfected into Marc-145 cells. After 24 h, the cells were infected with PRRSV at a MOI of 0.1 for 24 h. The mRNA and protein level of PRRSV orf7 was measured by qRT-PCR and Western blot, respectively. (**D**) The supernatants were collected at 24 h post-infection (hpi) for TCID50 assay. Data are presented as mean ± SEM pooled from one separated experiment; *n* ≥ 3 for each of the analyzed parameters. *, *p* < 0.05; **, *p* < 0.01.

The dual-luciferase assay demonstrated that miR2911 effectively inhibits luciferase activity by binding to the specific target on the PRRSV. This was supported by a significant decrease of over two-fold in luciferase activity in the miR2911 group compared to the NC group, providing initial evidence of the strong impact of the predicted miR2911 target region (Figure 3A). Overexpression of miR2911 in MARC-145 cells significantly inhibited PRRSV replication, leading to a significant decrease in PRRSV orf7 gene expression, viral titer, and viral N protein levels (Figure 3B–D).

## 4. Discussion

Honeysuckle is characterized as sweet and cold in nature, known for its ability to clear heat, remove toxins, and reduce inflammation and swelling. When paired with other traditional Chinese medicines, honeysuckle can effectively prevent and treat viral infections. Honeysuckle-derived miR2911 has been reported to play a significant role in antiviral activity by interacting with virus-specific genes, thus effectively inhibiting viral replication [19,20]. miR2911 exhibits stability in nature and acts through oral absorption by a passive mechanism [12,17]. Therefore, the regulatory effect of miR2911 on PRRSV was explored. miR2911 targets six regions of PRRSV, and these targeted regions are highly conserved. miR2911 demonstrates the ability to inhibit the replication of PRRSV by targeting its Nsp1 and Nsp2 regions.

Since the African swine fever (ASF) outbreak in 2018, the situation regarding the prevention and control of major viral infectious diseases in swine has become more severe in China. The persistence of old diseases alongside the emergence of new ones is the primary problem facing the swine industry at present. Viral infectious diseases often require immunization vaccines for prevention and control, as there are no specific therapeutic drugs available. Considering that honeysuckle-derived miR2911 has demonstrated efficacy in resisting influenza virus infection and novel coronaviruses, it was speculated that honeysuckle-derived miR2911 might have potential targets on PRRSV genes.

The targets of miR2911 on PRRSV virulence genes were successfully predicted using miRanda v1.0b software. miR2911 can target two genes of PRRSV, including four targeting regions on the PRRSV Nsp2 gene and two targeting regions on PRRSV orf1 (Nsp1 and Nsp2), playing a role in PRRSV mutation [21].

Mutated strains of PRRSV appear approximately every 8–10 years, with PRRSV Nsp2 playing a crucial role in mutation. Nsp2 exhibits deletions and mutations in different strains. Through sequence comparison analysis, it was found that the target sites of different PRRSV strains only have mutations in individual bases, and the target site of miR2911 on PRRSV Nsp2 avoids the deletion region. Therefore, honeysuckle-derived miR2911 has the potential to inhibit the replication of the current PRRSV epidemic strain.

Furthermore, by chemically synthesizing miR2911 homologs and its targeting region reporter plasmid, it was verified at the cellular level that miR2911 could target the PRRSV gene region to play an inhibitory role. miR2911 was demonstrated to significantly inhibit the replication of PRRSV through miR2911 overexpression and PRRSV infection assay. miR2911 is a class of RNAs that are stable and can act through absorption in the digestive tract of animals. It can be absorbed by the digestive tract of animals to function [17]. The next step is to validate the therapeutic effect of miR2911 on PRRSV-infected pigs through in vivo treatment by dosing miR2911 into pigs in an animal infection test. On this basis, miR2911 will be introduced into yeast, probiotics, etc., and the antiviral infection effect of miR2911 will be evaluated in animals by feeding the above transgenic strains to pigs, infecting them with viruses, and assessing detoxification and the degree of pathological damage in pigs. In addition, the miR2911 expression vector was constructed with pre-miR-339 as the backbone, and it was envisaged as follows (Figure S1). A miR2911 mature sequence replaced miR-339-5p, and then the sequence was inserted into the pcDNA3.1 vector polyclonal site. The overexpression plasmid can be achieved in the animal through the method of electrical stimulation by an intramuscular injection-assisted acupuncturist, or by the use of needleless injection. miR2911 exhibits a strong affinity for the relevant region of PRRSV Nsp2. Despite the presence of mutations at two to three loci, different strains show high homology in the targeting region of miR2911 on PRRSV. miR2911 targets a conserved region in the PRRSV gene, with high target homology observed among isolates from different time periods and regions. Therefore, honeysuckle-derived miR2911 has the potential to inhibit the replication of the current PRRSV epidemic strain. miR2911 significantly inhibits luciferase activity by binding to the PRRSV target. miR2911 overexpression and PRRSV infection experiments will be conducted to confirm its strong antiviral activity, providing a new avenue for the development of PRRSV antiviral drugs.

## Data Availability

Data sharing is not applicable to this article.

## Supplementary Materials

The following supporting information can be downloaded at: https://www.mdpi.com/article/10.3390/v16091350/s1, Figure S1: miR2911 eukaryotic expression design ideas; Table S1: miR2911 mimics sequence; Table S2: The sequence of miR2911 targeting PRRSV regions; Table S3: NC mimics sequence; Table S4: Realtime fluorescence quantitative PCR primer information.
